# How Well Are Socioeconomic Factors Associated With Improved Outcomes for Infants Diagnosed With Early Childhood Developmental Delay? An Observational Study

**DOI:** 10.3389/fped.2022.890719

**Published:** 2022-07-12

**Authors:** Fang Ji, Yao Sun, Yi Xu, Jian Tang, Jing Hu

**Affiliations:** ^1^Department of Rehabilitation Medicine, Children's Hospital of Nanjing Medical University, Nanjing, China; ^2^Member of Professional Committee of Rehabilitation Nursing of Nanjing Rehabilitation Medical Association, Nanjing, China

**Keywords:** early childhood developmental delay, parenting time, medical spending, distance to hospital, medical insurance coverage, health services accessibility

## Abstract

**Purpose:**

Early childhood developmental delay remains problematic worldwide in terms of weight and the five domains of child development, including gross motor, fine motor, cognition, language, and social domains. Based on the World Health Organization (WHO) guideline and the theoretical domain framework, this study identified five key socioeconomic factors, such as parenting time during hospitalization, parental educational level, medical spending, distance to hospital, and medical insurance coverage, to describe how these five factors are associated with improved outcomes of developmental quotient (DQ) values and the weight of infants in a tertiary hospital.

**Methods:**

In this prospective observational study, clinical and socioeconomic data were collected. Clinical data included the weight and DQ values of infants and other data relevant to the birth of infants. A National Developmental Scale was used to observe infants in five domains and calculate the DQ values of infants. These five domains include gross motor, fine motor, cognition, language, and social domains. Parenting time during hospitalizations was observed by a research nurse. Other socioeconomic factors were reported by parents and verified with system information.

**Results:**

A total of 75 infants' parents were approached, of which 60 were recruited. The age of infants ranged from 75 to 274 days at the first admission. Increments of their weight and DQ values improved from −0.5 to 2.5 kg and from −13 to 63, respectively. More than half of the parents (54.1%) were at the level of minimum secondary education although the results were not statistically significant. However, there was a positive correlation between weight improvement and parenting time during hospitalization (*r*(58) = 0.258, *p* < 0.05), medical spending (*r*(58) = 0.327, *p* < 0.05), distance to hospital (*r*(58) = 0.340, *p* < 0.01), but there was a negative association with medical insurance coverage (*r*(58) =-0.256, *p* < 0.05). There was also a significant relationship between the improved DQ value and distance to hospital (*r*(58)= 0.424, *p* < 0.01).

**Conclusion:**

Parenting time during hospitalization, medical spending, distance to hospital, and medical insurance coverage are important factors for early childhood developmental delay in relation to possible hospital intervention and improved accessibility to health services for families in rural areas. Therefore, changes in the current medical scheme are needed because a universal medical subsidy among regions will reduce the financial burden of families and provide families with more access to the necessary health services that their children need.

## Introduction

Early childhood development (ECD) has a lifelong purpose for both the individual and society. Improved outcomes of ECD contribute to children's future educational and career achievements (1, 2). Moreover, it is essential for the society to accumulate human capital and ultimately contribute to economic growth (3, 4). As infants' 1st year is the most biologically vulnerable year and sensitive to interventions, improving developmental outcomes in the 1st year is critical (5–10).

However, early childhood developmental delay (ECDD) remains problematic worldwide in terms of five main domains of childhood development, including gross motor, fine motor, cognition, language, and social domains (11–13). Globally, approximately 250 million children under the age of 5 years are at risk of not achieving their developmental milestones in the earliest years of life, for example, the global prevalence of autism spectrum disorders is ~3% (14, 15). In the USA, 14% of children under the age of 2 years are estimated to have a developmental delay (16), while approximately 43% of children in low-income countries did not reach their full potential during their ECD (17). In China, particularly in rural areas, children up to 3 years of age who have a developmental delay in at least one domain account for almost 60% of all rural children and approximately 42% of all children national wise (18), these developmental delays in infants are attributed to many factors, including pathological and non-pathological factors. Pathological factors that lead to ECDD include current and congenital medical conditions; non-pathological factors, such as the compromised quality of parenting time in some rural areas, where the interactions between parents and child are inadequate or unresponsive (17, 19, 20). Socioeconomic factors are important non-pathological factors identified in the literature and theoretical framework (5, 21, 22). While some pathological factors in relation to ECDD are well-researched (23, 24), there is little research on socioeconomic factors in relation to improving developmental outcomes of the 1st year of life. Therefore, based on the World Health Organization (WHO) guideline and the theoretical domains framework, five key socioeconomic factors are thus identified and discussed in this paper, including parenting time during hospitalization, parental educational level, medical spending, distance to hospital, and medical insurance coverage (5, 17, 21, 22). Parenting time during hospitalization in this study was considered different from parenting time in non-hospital settings in terms of knowledge, skills, practice, and attitude. Parenting time was influenced by healthcare professionals and medical information on a daily basis, therefore parenting time was defined and observed as time spent in the hospital setting in this study. Medical spending was defined as medical expenses that were relevant to the child's ECD condition on an annual basis.

This study aims to understand whether five key socioeconomic factors are associated with improved developmental outcomes after infants are diagnosed with ECDD in a tertiary children hospital in China. Subsumed in this aim, the following research questions are posed:

1. Is there a relationship between parenting time during hospitalization and the improvement in infant weight and/or developmental quotient (DQ)?
2. Is there a relationship between parent's educational level and the improvement in infant weight and/or DQ?
3. Is there a relationship between medical spending and the improvement in infant weight and/or DQ?
4. Is there a relationship between distance to hospital and the improvement in infant weight and/or DQ?
5. Is there a relationship between medical insurance coverage and the improvement in infant weight and/or DQ?

## Methods

In this prospective observational study, a structured observation tool was adopted, tested, and used to collect the demographic and clinical data of both infants and parents. Socioeconomic data were observed, collected, and verified with a medical team on the day of hospitalization. Thus, the time of observation started from the admission of infants to the ward until hospital discharge.

### Setting and Sample

This study was conducted in a 1,742-bed tertiary children hospital catering to both rural and urban areas. Approximately 69,100 hospital admissions and 29,100 surgeries are performed each year. In this study, the pediatric rehabilitation ward where infants were admitted in the hospital was purposively selected. The children admitted to this ward came from both urban and rural areas. Inclusion and exclusion criteria were applied: infants and parents are enrolled in the study in case the infants are under 1 years of age at the time of the first hospital admission due to ECDD, including single-domain and multi-domains developmental delays, and infants will require a minimum of two consecutive hospitalizations for rehabilitation. Based on the purpose of this study, infants were excluded from participation if they are born prematurely or have current medical conditions such as inherited disorders, diabetes, heart conditions, cancer, infections, and problems with hearing and vision; as these conditions that may result in ECDD are not the focus of the socioeconomic factors in this study. Parents or other legal guardians of children who were unable to provide informed consent were also excluded from participation. Given the presence of 371 potential eligible inpatients who met the inclusion criteria from 2015 to 2018 in this rehabilitation ward and the potential impact of COVID-19, convenient sampling is used, and the total sample size is 60 infants.

### Observational Tool Testing

A structured data collection tool of a National Developmental Scale for Children aged from 0 to 6 years underpinned by the Gesell Theory was adopted according to the hospital protocol (25), and a data dictionary containing conceptual and operational definitions was used in conjunction with the observation tool. This tool assessed a child's gross motor, fine motor, cognitive, language, and social domains. These five domains are important domains where the milestones are to be achieved during early infancy (26, 27). While some relationships are established among motor, cognitive, and language development (28), there are also many social milestones for babies to achieve during early infancy and social development plays an important role in the development of their brain neural network (29). The scoring system of the tool is to assess the developmental age of infants, which is based on the number of milestones achieved by infants at the time of assessment and using a formula to calculate DQ (30–32). The tool was piloted by two trained observers who are registered nurses and independently observed four infants, and interrater agreement of the tool was tested. Using this structured data collection tool, observations started when the infant was first admitted to the rehabilitation ward and ended at each hospital discharge. During hospitalization, the same observers approached parents for participation and collected data as well as documented explanatory field notes to give further description of case-specific nuances that might have a bearing on improved outcomes of the infant, for example, the family accessed the medical services in the urban area that is further away from the rural area in which they live. These field notes gave further context to support the structured observations in terms of understanding the variables, such as possible high medical spending and the low medical insurance coverage due to access to the medical services in urban areas.

### Ethic Clearance

Approval to conduct this study was granted by the institutional ethics committee at the hospital (IRB No. 202111123-1). Participants' legal guardians who were parents of infants in this study received written information explaining the aims, processes, risks, and benefits of the study, and informed consent was obtained for all observations and reconfirmed throughout the data collection period. Due to the continuing nature of observational studies, consent should always be reconfirmed and access should never be assumed without reconfirming consent.

### Measures and Data Collection

Observations were conducted from January 2019 to December 2020 for 24 months continuously. However, the study had an impact due to hospital restrictions in response to COVID-19. Patients including some potential eligible participants were not allowed to enter the hospital during several lockdowns and other restrictions among districts during the study period from 2019 to 2020. Therefore, the number of recruitments has been under the target, and the completion of the study has been delayed due to these impacts of COVID-19. The actual data collection period was about 9 months in total.

Infants and parents were observed by observers from the time of admission of the infant to the ward until hospital discharge. At each observation, observers used the structured data collection tool for developmental assessments and kept explanatory field notes to support structured observations of key independent variables, including parenting time during hospitalization, medical spending, physical distance to the hospital, medical insurance coverage, and parental educational level. Parenting time during hospitalization was observed and recorded, as parenting time during hospitalization was influenced in the ward context, where all parents received daily information on how to help their infants to improve in relation to ECDD, more specifically, parents' knowledge, skills, practices, and attitude were influenced in this context (26–28). The categorical variable included parents' educational level, which was dummy coded as “0” or “1” or “2” or “3.” Continuous variables included the following: parenting time during hospitalization, medical spending, physical distance to the hospital, and medical insurance coverage.

On the day of admission, infants were screened by ward observers according to inclusion and exclusion criteria and were recruited *via* convenience sampling. Infants' parents who consented were then allocated study numbers, and demographic data were collected. Consenting parents and their infants were followed by observers throughout the hospitalization. During hospitalization, the observation of the five domains of children development was undertaken after reconfirming the verbal consent from patients, as well as access to their electronic medical charts for data collection. Parenting time during hospitalization was recorded, and medical spending used on the child on an annual basis was reported by the family, as well as physical distance to the hospital and medical insurance coverage. The reported information will be verified with hospital records. According to the hospital protocol, infants and parents came for a routine check-up in 2 months after the second discharge, so their weight was collected at the clinic for this study.

### Data Analysis

Interrater reliability testing of the data collection tool was performed using Cohen's κ coefficient. Data were entered and cleaned prior to analysis using the statistical program, SPSS 27.0 for Windows software (SPSS, Inc., Chicago, Illinois). Descriptive statistics were used to describe the characteristics of infants and their clinical data at birth. Analyses were undertaken according to data level and distribution, and the results were reported as absolute (*n*) and relative (%), medians, and interquartile ranges (IQRs) in tables, as appropriate. Spearman's ρ test was used as a non-parametric alternative for ranked continuous variables. It is used in inferential analyses (hypothesis testing) to describe relationships between clinical and/or case factors and socioeconomic factors that contribute to improved outcomes of ECDD.

## Results

### Descriptive Results

A total of 75 infants' parents were approached, of which 60 infants were recruited. The age of infants at the first admission ranges from 75 to 274 days. Developmental outcomes of infants between the first and the second hospital discharge improved with increments of weight from −0.50 to 2.50 kg and DQ from −13 to 63, respectively. Kappa interrater agreement between two independent observers for the data collection tool was 98% (*p* < 0.0005), indicating a strong and highly significant degree of agreement between raters.

**Figure 1:**
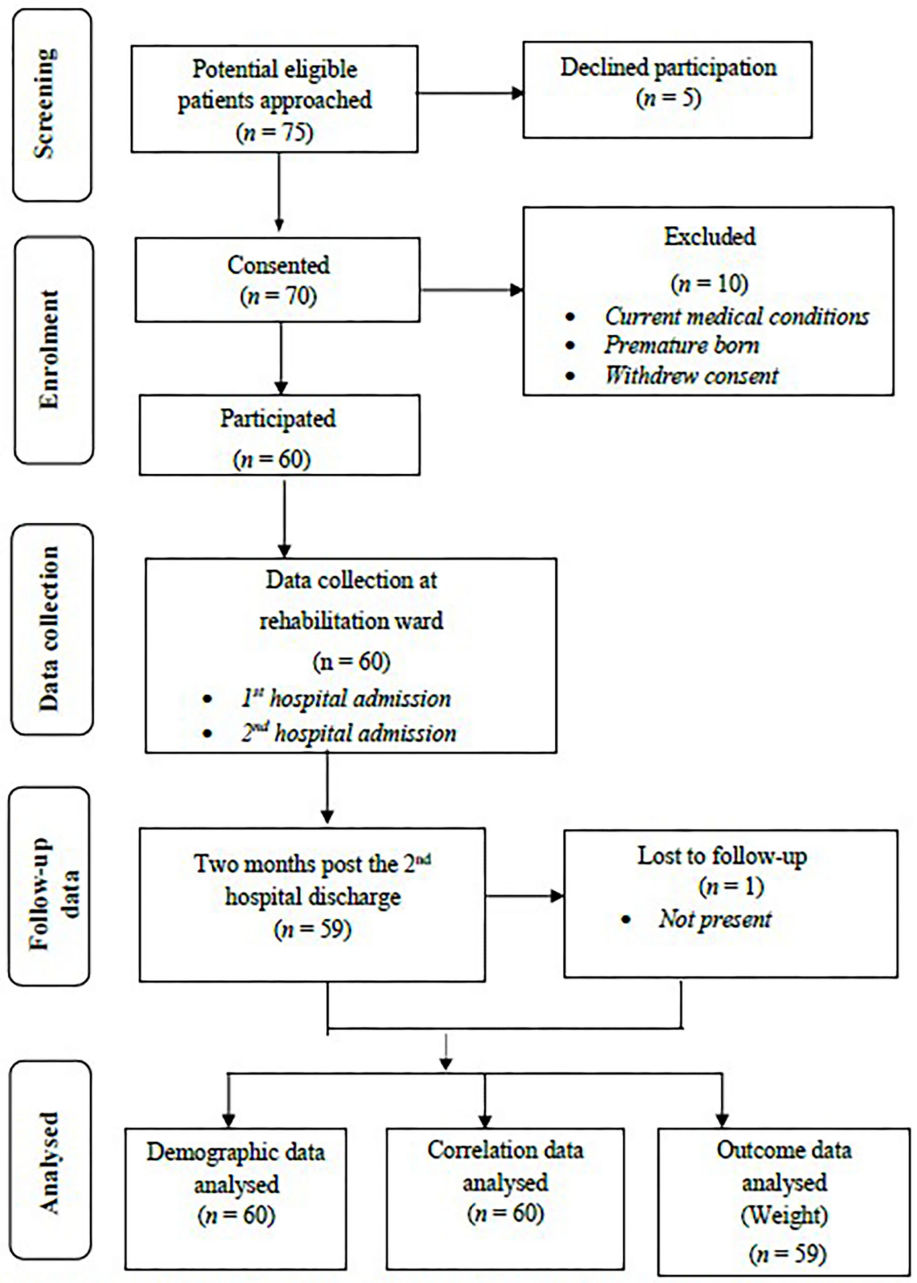
Flow diagram of data collection.

Figure 1 illustrates the flow of infants participated in this study. During the study, observers approached 75 parents of infants who met the inclusion criteria on the day of hospital admission for recruitment in the children rehabilitation ward or children clinic. Of the 75 patients who were screened and approached, 70 consented to participate in this study. Of the 70 patients who consented, 10 patients were excluded from this study because of their existing medical conditions, premature born status, and parental withdrawal of consent. The remaining 60 patients who consented continued their participation in this study. Observational data were collected based on 60 patients during infants' hospitalizations in the rehabilitation ward. Follow-up data related to the improvement in infant weight were collected in a clinic within 2 months after the second hospital discharge to capture potential further improved weight. One of the patients did not have weight data in 2 months' time as patients' weight was not presented to the clinic due to the distance from the hospital. However, their demographic and clinical data collected following enrolment were included in the descriptive and correlational analyses. Therefore, demographic data and relationships between variables were analyzed based on the 60 observed procedures, and outcome data in relation to weight improvement were analyzed based on 59 observed cases.

Table 1 presents the demographic, clinical, and socioeconomic factors of infants. Of the 60 infants included in the final analysis, the median age at the first hospital admission across the sample was 129 days (IQR = 49, range: 75–274 days), comprising 43/60 (71.7%) men, representing just under three-quarters of the total sample. The majority of infants (*n* = 53/60, 88.3%) were delivered on terms from 37 to 42 weeks. In relation to delivery, 31/60 (51.7%) infants were born spontaneously and 29/60 (48.3%) were born through cesarean section. Only 6/60 (10.0%) of infants' parents were conceived through *in vitro* fertilization and 9/60 (15%) of infants were born with low birth weight, while 39/60 (65.0%) of infants' parents were first-time parents.

**Table 1:** Demographic, clinical, and socioeconomic characteristics of patient samples (*n* = 60).

**Factors**	***n***	**%**
**Demographic factors**		
*Age*		
30–91 days	4	6.7
92–182 days	48	80.0
183–274 days	8	13.3
**Gender**		
Male	43	71.7
Female	17	28.3
**Term on delivery**		
28–36 weeks	6	10.0
37–42 weeks	53	88.3
> 42 weeks	1	1.7
**Delivery**		
Spontaneous vaginal delivery	31	51.7
Cesarean section	29	48.3
**Conceive**		
Spontaneous pregnancy	54	90.0
*In vitro* fertilization	6	10.0
**Weight at birth**		
≥2.5 kg	51	85.0
<2.5 kg	9	15.0
**First-time parent**		
First-time parent	39	65.0
Not first-time parent	21	35.0
**Clinical factors**		
**Weight changes between 1st admission and 2nd discharge**		
≤ 0 kg	10	16.7
>0 and ≤ 1 kg	30	50.0
>1 and ≤ kg	18	30.0
>2 kg	2	3.3
**Developmental Quotient changes between 1**st **admission and 2**nd **discharge**		
≤ 0	7	11.7
>0 and ≤ 20	33	55.0
>20 and ≤ 50	16	26.7
>50	4	6.7
**Months between two admissions**		
1–2 months	41	68.3
3–4 months	16	26.7
5–6 months	1	1.7
over 6 months	2	3.3
**Socioeconomic factors**		
**Parents' educational level**		
Primary education	9	15.0
Secondary education	33	55.0
Tertiary education	17	28.3
Above tertiary education	1	1.7
**Parenting time during hospitalizations**		
<5 h/day	6	10.0
5 h/day	3	5.0
6–10 h/day	11	18.3
24 h/day	40	66.7
**Distance to hospital**		
≤ 100 km or <2 h	30	50.0
≤ 200 km or 2–3 h	14	23.3
>200 km or 2.5 h	16	26.7
**Medical insurance coverage**		
10%	20	33.3
35%	18	30.0
65%	22	36.7
**Medical spending - percentage of family income**		
20%	5	8.3
30%	15	25.0
35%	1	1.7
40%	12	20.0
50%	12	20.0
60%	11	18.3
65%	3	5.0
70%	1	1.7
**Weight changes 2 months post second discharge** ^**∧**^	
≤ 0	21	35.0
>0 and ≤ 1	35	58.3
>1	3	5.0
Missing	1	1.7

*^∧^Missing data*

Clinical factors including infants' gross motor, fine motor, cognition, language, and social domains were observed in two hospital admissions, and their weight was also examined. In relation to improving the development of infants, after the second hospital admission, 1 or 2 month after the first hospital discharge (median = 63 days), half of the infants (30/60 outcomes, 50.0%) increased their weight 1 kg or under at the second discharge, and a third (20/60, 33.3%) increased their weight over 1 kg. However, 10 infants (10/60, 16.7%) lost weight or remained the same, while only seven infants (7/60, 11.7%) had no improved DQ. Nevertheless, 81.7% (49/60) of the total samples improved their DQ. Across the 60 observed samples, the median improved weight and the improved DQ were 1 kg (IQR = 1) and 18 (IQR = 17.3), respectively.

In socioeconomic factors, most parents had minimum secondary or tertiary education (33/60, 55.0% and 17/60, 28.3%, respectively) and most of the parenting time during hospitalization (40/60, 66.7%) was 24 h/day. While half of the samples (30/60, 50.0%) traveled to the hospital within 100 km, 23.3% (14/60) traveled within 100–200 km, and 26.7% (16/60) still had a distance of more than 200 km. More than one-third of the samples (22/60, 36.7%) had medical insurance coverage of 65%, while almost half of the samples (27/60, 45.0%) spent at least 50% of their family income on treating their infants' ECDD.

Of the 60 observed samples, 59 infants were followed up in a clinic within 2 months after the second hospital discharge and their weight was then collected. Most infants (38/60, 63.3%) increased their weight.

Table 2 reports improved outcomes of infant weight and DQ in two hospitalizations. Across 60 observations, infants' gross motor, fine motor, cognition, language, and social domains were observed and the DQ value was calculated. The predominant group (23/60, 38.3%) in the first hospital admission had a DQ ranging from 61 to 80, with none over 100. However, in the second hospital discharge, the predominant group (37/60, 61.7%) had improved DQ and shifted to a higher range from 81 to 100, as well as some (5/60, 8.3%) over 100. Furthermore, more than half of the infants (32/60, 53.3%) weighed at the first admission ranging from 7 to 8.9 kg and none over 11 kg, while at the second discharge, more infants (40/60, 66.7%) weighed within the same range and one infant weighed over 11 kg.

**Table 2:** Improved weight and DQ of infants during two hospitalizations (*n* = 60).

**Infants' improved weight and DQ**		**First hospital admission**		**Second hospital discharge**	
		***n***	**%**	***n***	**%**
**DQ**	21–40	6	10.0	1	1.7
	41–60	14	23.3	2	3.3
	61–80	23	38.3	15	25.0
	81–100	17	28.3	37	61.7
	101–120	-	-	5	8.3
**Weight**	5–6.9 kg	17	28.3	4	6.7
	7–8.9 kg	32	53.3	40	66.7
	9–10.9 kg	11	18.3	15	25.0
	11–12.9 kg	-	-	1	1.7

### Relationship Analyses

As shown in Table 3, Spearman's ρ test showed a positive correlation (_*r*(58)_ = 0.258, *p* < 0.05) between parenting time during hospitalization and improved outcomes of infant weight at the second hospital discharge across the sample (*n* = 60). Thus, the longer parenting time during hospitalization, the more weight the infants gained. There was also a moderate positive correlation between medical spending and improved outcomes of infant weight (*r*(58) = 0.327, *p* < 0.05). This finding suggests that the more medical spending, the better the infant weight outcomes were achieved.

**Table 3:** Correlation matrix.

**Variables**	***n***	**Improved weight**	**Improved DQ**	**Parenting time during hospitalization**	**Medical spending**	**Parent educational level**	**Distance to hospital**	**Medical insurance coverage**
Improved weight	60	1.000						
Improved DQ	60	0.544	1.000					
Parenting time during hospitalization	60	0.047 *	0.855	1.000				
Medical spending	60	0.011*	0.150	0.002**	1.000			
Parent educational level	60	0.225	0.116	0.073	0.001	1.000		
Distance to hospital	60	0.008**	0.001**	0.011*	0.000**	0.338	1.000	
Medical insurance coverage	60	−0.048*	−0.196	0.030*	−0.000**	0.009**	−0.000**	1.000

^*^ p <0.05.

^**^ p <0.01.

The results also show distance to hospital was moderately positively associated with improved weight and improved DQ (*r*(58) = 0.340, *p* < 0.01 and *r*(58) = 0.424, *p* < 0.01, respectively), while medical insurance coverage was negatively associated with improved outcomes of infant weight (*r*(58) = −0.256, *p* < 0.05). This finding suggests that further away from the hospital where the family lives, the better the improvement in infant weight and DQ, and infant weight also improved when the medical insurance coverage was lower.

Nevertheless, there were no statistically significant correlations between improved outcomes of DQ and parenting time of infants during hospitalization, medical spending, or medical insurance coverage, and the parent educational level was statistically non-significantly associated with improved outcomes of the weight or DQ of infants.

## Discussion

Three important results are highlighted in this study. Firstly, the improved health outcomes of the weight and DQ of infants have been seen in most infants after the first hospital discharge. This reinforces that the 1st year of the life is critical for infants who are diagnosed with ECDD to access the medical resources and achieve the improved health outcomes. In this study, it is also noted that the majority of infants were boys, while the broader literature has not provided the information of participants' gender.

Secondly, there were significant relationships between parenting time during hospitalizations, medical spending, and improved outcomes of infant weight. This finding signifies the unique facilitator of parenting time in relation to the improved health outcomes of infants in a hospital setting where the quality of parenting time is influenced by healthcare professionals and daily educational information in terms of knowledge, skills, practices, and attitudes (20, 33–35). This also indicates that medical spending is necessary for improving health outcomes of developing infants. This finding is consistent with findings in the broader literature (36–38), and the theoretical frameworks that informed the rationale for identifying key socioeconomic factors for this study (21, 36). Future research may need to further unpack the observed domains of DQ and examine their associations individually with improved health outcomes to inform evidence-based interventions for ECDD in a hospital setting.

Thirdly, there was a positive correlation between distance to hospital and improved outcomes of the weight and DQ of infants. Longer distance or further away from the hospital, the more weight or DQ increments the infants achieved. This seems difficult to understand, as well as a negative correlation between medical insurance coverage and improved infant weight outcomes. The lower medical insurance coverage, the more weight increments the infants achieved. Research shows that living further away from the hospital in China means living in a zone far from necessary resources, for example, there is a low percentage of medical insurance coverage in the rural area, therefore inequities in accessing the medical resources are increased and infants with potential ECDD may miss out their early diagnosis and treatment (39). In this study, half of the samples were living further away from the hospital, and more than half of the samples had low medical insurance coverage. The parents of these samples had to manage to access medical resources through either a recent government-supported referral system between hospitals for higher medical insurance coverage, or a self-funded referral with low medical insurance coverage. These parents moved to another city where the hospital was located. As these parents managed to access to medical resources by paying a significant amount of money, there were also significant improvements in health in relation to weight and/or DQ increments of infants. Therefore, the results indicated that in these samples, distance to hospital was positively associated with improved weight and DQ, while medical insurance coverage was negatively associated with improved weight. This is consistent with other findings from previous research (39). Therefore, increasing accessibility to medical resources in terms of physical distance and financial support can significantly benefit the health of infants for families living in rural areas.

Currently, there is little research that specifically examine the effect of the intervention of parenting time at the hospital on improving infants' development during their 1st year of life. The WHO guidelines strongly recommend providing infants with responsive care and applying parenting intervention for ECDD (5). The guidelines also recommend achieving greater outcomes for ECD by not only using responsive care or parenting intervention alone (5). However, there is a lack of clarity around the implementation of this guideline. While the need for improving the outcomes of the weight and DQ of infants is prioritized, the definition of parenting intervention in the context is still vague, which demonstrates the difficulties in improving the hospital parenting intervention program and highlights the existing challenges in developing operationalized guidelines for its use. In the current study, we observed the quality interactions between parents and infants measured in time form. However, we need to acknowledge that the definition of parenting time during hospitalization in this study has its limitations, as not all quality parenting times have been observed and some quality parenting time at hospital may not necessarily be attributed to hospital influence. Instead, it may be attributed to the prior knowledge and experience, such as second-time parents who already obtained relevant knowledge and/or experience. Therefore, we cannot simply claim a statistic non-significant relationship between parenting time during hospitalization and the DQ of infants. Defining quality parenting time more cautiously needs to be considered in future research.

This study had a theoretical basis and was based on international clinical practice guidelines and other best available evidence around ECD in the literature. Data were analyzed against socioeconomic risk factors identified in the literature. Compared to existing research, the age of infants has been accurately recorded at different observational time points, which provide benefits of a better understanding of the samples' characteristics. The data collection tool was adopted based on the Gesell Theory and the hospital protocol, and was pilot tested by two independently trained raters. Data collection in this study encompassed the entire patient's hospitalization journey, commencing at the ward admission and being collected daily with a follow-up in 2 months after the second hospital discharge, which enabled a more comprehensive collection of data in relation to the improvements in the development of infants.

However, this study was conducted in a single hospital site, which might be atypical in relation to other hospitals in the region. The use of convenient sampling methods may give rise to selection bias. There is a possibility of the Hawthorn effect as infants' parents may change their caring behavior due to the awareness of being observed. However, with the prolonged presence of the observer, the Hawthorn effect is likely to be somewhat mitigated as parents become accustomed to the presence of the observer (40). Further, the potential observation of some information was lost due to workflow issues, but these instances were documented as field notes to give more detailed information on these types of situations. Finally, outcome data related to post-discharge DQ were missed, as infants only presented to the clinic and did not present to the rehabilitation ward where the observation of five domains could take place; this study had limitations of time and budgetary resources for additional post-discharge follow-up. A larger multisite study could address some of these limitations.

## Conclusions

Based on the five key socioeconomic factors identified in the literature, this observational study of improved outcomes of the weight and DQ of infants in relation to ECDD confirms that: (1) parenting time during hospitalization and medical spending is critical for infants diagnosed with ECDD to improve their health outcomes during early infancy; (2) the current medical subsidy scheme varies among cities or regions, which decreases accessibility to the necessary health services the child needs. The socioeconomic factors of distance to hospital and medical insurance coverage can significantly improve the development of infants, especially for families living in a distal area; (3) there is a lack of research to guide how to transit current knowledge into parenting practice and use hospital-based parenting intervention in relation to ECDD; and (4) further research on how to define quality parenting time and how these five key socioeconomic factors interact with each other in clinical practice may improve infant health outcomes in relation to ECDD in the future.

## Data Availability Statement

The raw data supporting the conclusions of this article will be made available by the authors, without undue reservation.

## Ethics Statement

The studies involving human participants were reviewed and approved by Children's Hospital of Nanjing Medical University. Written informed consent to participate in this study was provided by the participants' legal guardian/next of kin.

## Author Contributions

JH, FJ, and JT contributed to conception and design of the study. FJ collected and cleaned the data and wrote the first draft of the manuscript. YS and YX verified the dataset. JH and FJ performed the statistical analysis. All authors wrote sections of the manuscript and contributed to manuscript revision, read, and approved the submitted version.

## Funding

The author declares that the open access publication fees are provided by the study participated institution.

## Conflict of Interest

The authors declare that the research was conducted in the absence of any commercial or financial relationships that could be construed as a potential conflict of interest.

## Publisher's Note

All claims expressed in this article are solely those of the authors and do not necessarily represent those of their affiliated organizations, or those of the publisher, the editors and the reviewers. Any product that may be evaluated in this article, or claim that may be made by its manufacturer, is not guaranteed or endorsed by the publisher.
