# Supplementary material for: How Well Are Socioeconomic Factors Associated With Improved Outcomes for Infants Diagnosed With Early Childhood Developmental Delay? An Observational Study
Source: Front Pediatr. 2022 Jul 12;10:890719. doi: 10.3389/fped.2022.890719 (PMC9315099; doi:10.3389/fped.2022.890719)
Supplement: Supplementary file 1 [file Data_Sheet_1.docx]

Table 1: Demographic, Clinical and Socioeconomic Characteristics of the Patient Samples (*n = 60*)

| Factors | n | % |
| --- | --- | --- |
| Demographic factors |  |  |
| *Age* |  |  |
| 30 - 91 days | 4 | 6.7 |
| 92 - 182 days | 48 | 80.0 |
| 183 - 274 days | 8 | 13.3 |
| *Gender* |  |  |
| Male  Female | 43  17 | 71.7  28.3 |
| *Term on delivery* |  |  |
| 28 - 36 weeks | 6 | 10.0 |
| 37 - 42 weeks | 53 | 88.3 |
| > 42 weeks | 1 | 1.7 |
| *Delivery* |  |  |
| Spontaneous vaginal delivery | 31 | 51.7 |
| Caesarean section | 29 | 48.3 |
| *Conceive* |  |  |
| Spontaneous pregnancy | 54 | 90.0 |
| In vitro fertilization | 6 | 10.0 |
| *Weight at birth* |  |  |
| ≥ 2.5 kilograms | 51 | 85.0 |
| < 2.5 kilograms | 9 | 15.0 |
| *First time parent* |  |  |
| First-time parent | 39 | 65.0 |
| Not first-time parent | 21 | 35.0 |
| Clinical factors |  |  |
| *Weight changes between 1^st^ admission and 2^nd^ discharge* |  |  |
| ≤ 0 kilograms | 10 | 16.7 |
| > 0 and ≤ 1 kilograms | 30 | 50.0 |
| > 1 and ≤ 2 kilograms | 18 | 30.0 |
| > 2 kilograms | 2 | 3.3 |
| *Developmental Quotient changes between 1^st^ admission and 2^nd^ discharge* |  |  |
| ≤ 0 | 7 | 11.7 |
| > 0 and ≤ 20 | 33 | 55.0 |
| > 20 and ≤ 50 | 16 | 26.7 |
| > 50 | 4 | 6.7 |
| *Months between two admissions* |  |  |
| 1 - 2 months | 41 | 68.3 |
| 3 - 4 months | 16 | 26.7 |
| 5 - 6 months | 1 | 1.7 |
| over 6 months | 2 | 3.3 |
| Socioeconomic factors |  |  |
| *Parents' educational level* |  |  |
| Primary education | 9 | 15.0 |
| Secondary education | 33 | 55.0 |
| Tertiary education | 17 | 28.3 |
| Above tertiary education | 1 | 1.7 |
| *Parenting time during hospitalisations* |  |  |
| < 5 hours/day | 6 | 10.0 |
| 5 hours/day | 3 | 5.0 |
| 6-10 hours/day | 11 | 18.3 |
| 24 hours/day | 40 | 66.7 |
| *Distance to hospital* |  |  |
| ≤ 100 kilometres or < 2 hours | 30 | 50.0 |
| ≤ 200 kilometres or 2-3 hours | 14 | 23.3 |
| > 200 kilometres or 2.5 hours | 16 | 26.7 |
| *Medical insurance coverage* |  |  |
| 10% | 20 | 33.3 |
| 35% | 18 | 30.0 |
| 65% | 22 | 36.7 |
| *Medical spending - percentage of family income* |  |  |
| 20% | 5 | 8.3 |
| 30% | 15 | 25.0 |
| 35% | 1 | 1.7 |
| 40% | 12 | 20.0 |
| 50% | 12 | 20.0 |
| 60% | 11 | 18.3 |
| 65% | 3 | 5.0 |
| 70% | 1 | 1.7 |
| *Weight changes two months post second discharge* ^ | |  |
| ≤ 0 kilograms | 21 | 35.0 |
| > 0 and ≤ 1 kilograms | 35 | 58.3 |
| > 1 kilograms | 3 | 5.0 |
| Missing | 1 | 1.7 |
| ^ Missing data. |  |  |
|  |  |  |

Table 2: Infants’ Improved Weight and DQ during two hospitalisations (*n = 60)*

| Infants' improved  weight and DQ | | First hospital admission | | Second hospital  discharge | |
| --- | --- | --- | --- | --- | --- |
|  |  | n | % | n | % |
| DQ | 21 - 40 | 6 | 10.0 | 1 | 1.7 |
|  | 41 - 60 | 14 | 23.3 | 2 | 3.3 |
|  | 61 - 80 | 23 | 38.3 | 15 | 25.0 |
|  | 81 - 100 | 17 | 28.3 | 37 | 61.7 |
|  | 101 - 120 | - | - | 5 | 8.3 |
| Weight | 5 - 6.9 kgs | 17 | 28.3 | 4 | 6.7 |
|  | 7 - 8.9 kgs | 32 | 53.3 | 40 | 66.7 |
|  | 9 - 10.9 kgs | 11 | 18.3 | 15 | 25.0 |
|  | 11 - 12.9 kgs | - | - | 1 | 1.7 |

Table 3: Correlation Matrix

| Variables | n | Improved weight | Improved DQ | Parenting time during hospitalisation | Medical spending | Parent educational level | Distance to hospital | Medical insurance coverage |
| --- | --- | --- | --- | --- | --- | --- | --- | --- |
| Improved weight | 60 | 1.000 |  |  |  |  |  |  |
| Improved DQ | 60 | 0.544 | 1.000 |  |  |  |  |  |
| Parenting time during hospitalisation | 60 | 0.047 * | 0.855 | 1.000 |  |  |  |  |
| Medical spending | 60 | 0.011* | 0.150 | 0.002** | 1.000 |  |  |  |
| Parent educational level | 60 | 0.225 | 0.116 | 0.073 | 0.001 | 1.000 |  |  |
| Distance to hospital | 60 | 0.008** | 0.001** | 0.011* | 0.000** | 0.338 | 1.000 |  |
| Medical insurance coverage | 60 | -0.048* | -0.196 | 0.030* | -0.000** | 0.009** | -0.000** | 1.000 |

* p < 0.05

** p < 0.01
